# Regulation of chemicals demands assessment of risks rather than identification of hazards only

**DOI:** 10.1007/s00204-026-04307-0

**Published:** 2026-02-07

**Authors:** P. Marx-Stoelting, V. Ritz, M. Herzler, C. Kneuer, K. Aiello-Holden, S Brescia, S. E. Escher, S. Feustel, E. Fritsche, J. Gebel, E. F. Kenny, R. Landsiedel, P. Sanders, M. Schwarz, M Streitz, E Testai, B. van Ravenzwaay, W. Waetjen, K. Wend, M. Wilks, A. Luch, T. Tralau, A. Hensel

**Affiliations:** 1 German Federal Institute for Risk Assessment (BfR), Berlin, Germany; 2 Health and Safety Executive (HSE), Chemicals Regulation Division (CRD), Merseyside, UK; 3 Fraunhofer ITEM, Hannover, Germany; 4 Swiss Centre for Applied Human Toxicology (SCAHT), University of Basel, Basel, Switzerland; 5 University Hospital Bonn, Institute for Hygiene and Public Health, Bonn, Germany; 6 German Society for Toxicology (GT), Mainz, Germany; 7 Institute for Pharmacology and Toxicology, Freie Universität Berlin, Berlin, Germany; 8 French National Agency for Food, Environmental and Occupational Health and Safety, Paris, France; 9 Department for Experimental and Clinical Pharmacology and Pharmacogenomics, Eberhard Karls University, Tübingen, Germany; 10 Friedrich-Löffler-Institute, Riems, Germany; 11 Formerly Instituto Superiore di Sanità, Environment and Health Dept., Rome, Italy; 12 University of Wageningen, Wageningen, The Netherlands; 13 Institute for Agriculture and Food Science, Martin-Luther-Universität Halle-Wittenberg, Halle, Germany; 14 German Federal Ministry for Agriculture, Food and Rural Environments, Berlin, Germany; 15 Department of Pharmaceutical Sciences, University of Basel, Basel, Switzerland

## Abstract

This position paper was collaboratively written during the international expert symposium “EU Chemicals Assessment - Risk- or Hazard-based?” that was organised by the German Federal Institute for Risk Assessment (BfR) in Berlin on November 27th and 28th 2025. Twenty experts from several institutions and European countries considered the scientific merits of both hazard-based and risk-based approaches to chemical safety assessment. While hazard information is essential, it does not reflect real-world exposure conditions that determine the likelihood of harm. On balance, we support a risk-based approach because it enables more proportionate, transparent and scientifically grounded regulatory decisions.

## Background

1. Man-made chemicals are crucial for the survival and well-being of humanity. For example, drugs and medical products as well as biocidal products, such as disinfectants, are saving thousands of lives every single day. Agrochemicals are important to feed a human population of eight billion or more. The construction of buildings or manufacturing of clothing that protect humans from environmental stressors depends critically on the availability of industrial chemicals. Furthermore, the production of energy, regardless of the source, is not possible without chemicals. This is also true for transportation and many other societal activities.
2. However, chemicals, man-made or natural, may also have hazardous properties. Consequently, chemicals may contribute to the development of acute or chronic diseases. In fact, all substances can be hazardous: whether or not there is a risk of harm to humans depends on the dose which is largely determined by factors such as the route, frequency, timing and duration of exposure and biological processes such as uptake, metabolism and elimination of the chemical. Other biological factors, including the specific mode of action, potency and individual susceptibility, play an important role, too.
  Identification and characterisation of such hazards is a key element of toxicology as a scientific discipline.
3. Establishing health-based guidance values (i.e., safe levels of exposure) to prevent hazards from adversely affecting human health is a key task of regulatory toxicology and constitutes an important part of chemical regulations.
4. In the context of chemical safety, the term ‘risk’ refers to the nature and severity of a hazard in combination with its likelihood of occurrence depending on the level of exposure (for a list of definitions see Box 1).

## Chemical regulation

5. Worldwide chemical regulations have successfully implemented the concept of risk assessment. This means that, to protect human health (and the environment), measures are implemented to reduce chemical exposure to a level below which it does not induce harm on the basis of best available knowledge. Rather than banning a chemical (or a group of chemicals as a whole), a limit value is identified below which its use is considered safe, allowing society to benefit from the chemical while being adequately protected at the same time.
6. Similar to chemical regulations on the global level, key parts of EU chemical regulations, including the REACH Regulation (Reg. (EC) No 1907/2006), the Plant Protection Products Regulation (Reg. (EC) No 1107/2009), the Biocidal Products Regulation (Reg. (EU) No 528/2012), the CLP Regulation (Reg. (EC) No 1272/2008), the Water Framework Directive (2000/60/EC), the Food Contact Material Regulation (Reg. (EC) No 1935/2004), Cosmetics Regulation (Reg. (EC) No 1223/2009), Pharmaceuticals Regulation (Reg. (EC) No 726/2004) or the Contaminants Regulation (Reg. (EU) No 2023/915) aim to protect human health and the environment from exposures to chemicals at levels that pose a risk.
7. Classification, labelling and packaging (CLP) of chemical substances under Regulation 1272/2008, where applicable, is part of the regulatory process. For example, depending on their hazards, chemicals may be classified as acutely toxic, sensitising, causing specific target organ toxicity (STOT) or being carcinogenic (C), mutagenic (M), toxic to reproduction (R) or endocrine disrupting (E) (summarised as CMRE). The aim is to ensure the safe use, handling, transport, and disposal of chemicals by providing a standardized approach to communicating hazards.
8. However, while most of the regulations mentioned under point 6 mainly use and apply the concept of risk, some among them ban chemicals or severely restrict their use based on possessing a specific hazard only (like CMRE), referred to as cut-off criteria. These cut-off criteria do not consider limit values and whether specific uses of these chemicals are associated with a risk.
9. Hazard-based cut-off criteria were introduced with good intentions in the area of plant protection products and biocides to drive innovation and improve human health. However, within a decade of experience, it has become clear that the intentions were not entirely achieved, and at the same time new challenges have emerged.
  As an example, substances such as ethanol or iodine, both of which have beneficial effects on human health as disinfectants, can safely be used. However, if they were classified for CMRE, these substances would be banned from use under the Biocidal Products Regulation. Meanwhile they could still be used as industrial chemicals under the REACH regulation or continue to be an integral constituent of the human diet.
10. To resolve such inconsistencies in the context of the ‘One Substance One Assessment’ (OSOA) principle, a purely hazard-based approach resulting in regulatory measures such as cut-off criteria is not appropriate. Such measures are quite unique to the EU market and have not been adopted worldwide. They hinder the beneficial safe use of those chemicals regulated by hazard-based cut-off criteria and obstruct global harmonisation of chemical regulation, creating barriers to innovation, trade and manufacturing.
11. There is a need in the EU to ensure continuation and enhancement of policies for the protection of human health and the environment from the potential harmful effects of chemical substances. This paper aims to provide a scientific recommendation to policy makers on the use of risk assessment as the key principle to avoid adverse effects from chemicals in humans while regulating chemicals in a way that still allows their safe use for the benefit of society.

## Thresholds

12. Chemicals can have a similar hazard but vary in potency by many orders of magnitude.
13. One of the key considerations associated with hazard-based regulation is the assumption that no dose threshold of adversity exists for certain hazards.
14. While there is scientific and societal consensus that most (if not all) potentially adverse effects do have a threshold below which regulation is not required or meaningful, the debate particularly focuses on two endpoints: mutagenicity and endocrine disruption.
15. However, besides theoretical considerations, convincing evidence is accumulating that even for compounds with mutagenic or endocrine disruptive properties, thresholds of adversity can be identified.
16. Furthermore, even under the assumption of non-threshold effects (or if thresholds exist, but cannot be determined experimentally), risk-based regulation is possible based on the likelihood of risk, as demonstrated for example by the way in which exposure to genotoxic carcinogens is addressed under REACH or other international regulations outside of the EU.

## Research needs and next generation risk assessment (NGRA)

17. There are still many substances where data for risk assessment are insufficient, and it does not seem efficient or even realistic to obtain a large part of these data by using traditional animal testing. Strategies based on New Approach Methodologies (NAMs) will likely contribute to closing these data gaps. Moreover, in the context of the EU Commission’s roadmap for phasing out animal testing for chemical safety assessments, changing the testing paradigm towards non-animal approaches has been proposed.
18. The data obtained by NGRA will facilitate moving away from purely hazard-based regulations, as NGRA is focussing on mechanistic data from human cells or test systems, integrating elements such as toxicokinetics, metabolism, dose-responses and exposure levels.
19. In contrast, classification and labelling under the CLP regulation currently heavily rely on animal testing. NAM-based classification may therefore require adaptation of the current Globally Harmonized System (GHS) on classification and labelling and CLP criteria, by integrating data informing on potencies, internal exposures and molecular pathways affected.
20. In the context of risk assessment, the concept of effect threshold needs further investigation and, given the probabilistic nature of risk as such, a paradigm shift from deterministic towards probabilistic risk assessment should be advanced.
21. Moreover, in risk assessment, exposure plays a central role. Thus, data allowing for a better understanding of exposure and use patterns are essential.
22. As humans are generally exposed to numerous substances, the issue of mixture effects also has to be considered. Methodologies for risk assessment of mixtures, whether intentional or coincidental, have been developed and applied without the need of an exclusively hazard-based approach.

## Conclusion

It is the role and responsibility of scientists to ensure that risk management decisions on chemicals are guided by the best available scientific knowledge and methodology.

Purely hazard-based approaches such as exclusion or cut-off criteria ignore an important and central part of the scientific knowledge available, thereby lacking sufficient scientific justification and often do not consider the full complexity of chemical use and regulation.

Overall, this document strongly supports risk assessment as the scientific gold standard to protect humans from harmful effects of chemicals.

Since regulatory practice, particularly in the area of biocides and plant protection products, is partly hazard-based due to the cut-off criteria, a change towards risk-based approaches requires an update of the legal provisions and a strengthening of exposure science and assessment.

## Literature and background

For a general overview on the debate risk versus hazard please see e.g., Herzler et al. (2021), Barile et al. (2021), Boobis et al. (2016) or Doe et al. (2021).

General definitions can be obtained from IPCS (2004). For the debate on endocrine disruptors please refer to e.g., Testai et al. (2013) or Solecki et al. (2017).

For questions regarding threshold please check Marx-Stoelting et al. (2014), Brescia (2020), Choi et al. (2024), Koch et al. (2025) or Menz et al. (2023).

For NAM and NGRA or mixtures please refer to for example Tralau et al. (2021), Sewell et al. (2024) or Marx-Stoelting et al. (2023).

Box with definitions (according to IPCS 2004)

1. Risk: The probability of an adverse effect in an organism, system, or (sub)population caused under specified circumstances by exposure to an agent.
  Risk assessment: A process intended to calculate or estimate the risk to a given target organism, system, or (sub)population, including the identification of attendant uncertainties, following exposure to a particular agent, taking into account the inherent characteristics of the agent of concern as well as the characteristics of the specific target system.
  The risk assessment process includes four steps: hazard identification, hazard characterisation (related term: dose–response assessment), exposure assessment, and risk characterisation. It is the first component in a risk analysis process.
  Risk analysis: A process for controlling situations where an organism, system, or (sub)population could be exposed to a hazard. The risk analysis process consists of three components: risk assessment, risk management, and risk communication.
2. Hazard identification: The identification of the type and nature of adverse effects that an agent has an inherent capacity to cause in an organism, system, or (sub)population. Hazard identification is the first stage in hazard assessment and the first of four steps in risk assessment.
3. Hazard characterisation: The qualitative and, wherever possible, quantitative description of the inherent property of an agent or situation having the potential to cause adverse effects. This should, where possible, include a dose–response assessment and its attendant uncertainties.
  Hazard characterisation is the second stage in the process of hazard assessment and the second of four steps in risk assessment.
4. Exposure assessment: Evaluation of the exposure of an organism, system, or (sub)population to an agent (and its derivatives). Exposure is defined as the concentration or amount of a particular agent that reaches a target organism, system, or (sub)population in a specific frequency for a defined duration. Exposure scenario is a set of conditions or assumptions about sources, exposure pathways, amounts or concentrations of agent(s)involved, and exposed organism, system, or (sub)population (i.e., numbers, characteristics, habits) used to aid in the evaluation and quantification of exposure(s) in a given situation.
  Exposure assessment is the third step in the risk assessment process.
5. Risk characterisation: The qualitative and, wherever possible, quantitative determination, including attendant uncertainties, of the probability of occurrence of known and potential adverse effects of an agent in a given organism, system, or (sub)population, under defined exposure conditions
  Risk characterisation is the fourth step in the risk assessment process
6. Adversity: A change in morphology, physiology, growth, reproduction, development or lifespan of an organism which results in impairment of functional capacity or impairment of capacity to compensate for additional stress or increased susceptibility to the harmful effects of other environmental influences
